# GQ-16, a TZD-Derived Partial PPARγ Agonist, Induces the Expression of Thermogenesis-Related Genes in Brown Fat and Visceral White Fat and Decreases Visceral Adiposity in Obese and Hyperglycemic Mice

**DOI:** 10.1371/journal.pone.0154310

**Published:** 2016-05-03

**Authors:** Michella S. Coelho, Caroline L. de Lima, Carine Royer, Janaina B. Silva, Fernanda C. B. Oliveira, Camila G. Christ, Sidney A. Pereira, Sonia N. Bao, Maria C. A. Lima, Marina G. R. Pitta, Ivan R. Pitta, Francisco A. R. Neves, Angélica A. Amato

**Affiliations:** 1 Laboratório de Farmacologia Molecular, Faculdade de Ciências da Saúde, Universidade de Brasília, Brasília, Brazil; 2 Departamento de Biologia Celular, Instituto de Ciências Biológicas, Universidade de Brasília, Brasília, Brazil; 3 Núcleo de Pesquisa em Inovação Terapêutica, Universidade Federal de Pernambuco, Recife, Brazil; Nihon University School of Medicine, JAPAN

## Abstract

**Background:**

Beige adipocytes comprise a unique thermogenic cell type in the white adipose tissue (WAT) of rodents and humans, and play a critical role in energy homeostasis. In this scenario, recruitment of beige cells has been an important focus of interest for the development of novel therapeutic strategies to treat obesity. PPARγ activation by full agonists (thiazolidinediones, TZDs) drives the appearance of beige cells, a process so-called browning of WAT. However, this does not translate into increased energy expenditure, and TZDs are associated with weight gain. Partial PPARγ agonists, on the other hand, do not induce weight gain, but have not been shown to drive WAT browning. The present study was designed to investigate the effects of GQ-16 on BAT and on browning of WAT in obese mice.

**Methods:**

Male Swiss mice with obesity and hyperglycemia induced by high fat diet were treated with vehicle, rosiglitazone (4 mg/kg/d) or the TZD-derived partial PPARγ agonist GQ-16 (40 mg/kg/d) for 14 days. Fasting blood glucose, aspartate aminotransferase, alanine aminotransferase and lipid profile were measured. WAT and brown adipose tissue (BAT) depots were excised for determination of adiposity, relative expression of *Ucp-1*, *Cidea*, *Prdm16*, *Cd40 and Tmem26* by RT-qPCR, histological analysis, and UCP-1 protein expression analysis by immunohistochemistry. Liver samples were also removed for histological analysis and determination of hepatic triglyceride content.

**Results:**

GQ-16 treatment reduced high fat diet-induced weight gain in mice despite increasing energy intake. This was accompanied by reduced epididymal fat mass, reduced liver triglyceride content, morphological signs of increased BAT activity, increased expression of thermogenesis-related genes in interscapular BAT and epididymal WAT, and increased UCP-1 protein expression in interscapular BAT and in epididymal and inguinal WAT.

**Conclusion:**

This study suggests for the first time that a partial PPARγ agonist may increase BAT activity and induce the expression of thermogenesis-related genes in visceral WAT.

**General Significance:**

These findings suggest that PPARγ activity might be modulated by partial agonists to induce WAT browning and treat obesity.

## Introduction

Obesity and type 2 diabetes are currently major health problems worldwide. The rate at which new cases are emerging and the significant risk of both morbidity and mortality stemming from its long-term vascular complications pose great health, social and economic challenges worldwide. In this scenario, the identification of adipose tissue as a key regulator of energy homeostasis has placed the adipocyte as an important focus of interest for the development of novel therapeutic strategies to treat metabolic disease.

Adipose tissue is traditionally classified as white adipose tissue (WAT) and brown adipose tissue (BAT). WAT is an endocrine organ well known by its ability to store chemical energy in the form of triglycerides, whereas BAT, although sharing the ability to synthetize lipids, is specialized in dissipating energy as heat in a process so-called adaptive thermogenesis, by means of the action of uncoupling protein 1 (UCP-1) [1]. Human adults were considered devoid of functional BAT depots until recently, when its presence was demonstrated by functional imaging procedures and by the expression of UCP-1 [2–4]. Interestingly, the amount of BAT was closely associated with indicators of metabolic health, such as lower body mass index and blood glucose levels [2–3]. A third type of adipocyte, so-called “beige” or “brite”, was described in rodents as an inducible cell type in WAT depots, in a process named browning of white fat. These cells share morphological features with brown adipocytes, express markers of thermogenesis, such as UCP-1, and display full thermogenic capacity upon stimuli such as cold and β3-adrenergic signaling [5]. Accordingly, they have been shown to protect mice against diet-induced obesity [6–7]. Beige adipocytes have a unique genetic signature, and have been identified in the cervical and interscapular regions of human adults, locations where genuine BAT is found in human infants [5]. In fact, it was shown that human adults have fat depots with both brown and beige features in these locations [8–11]. These findings suggest that data on beige adipocytes may be translated from rodents to humans and have rendered this type of adipocyte as an attractive target to treat obesity. There has thus been an intense effort to identify factors that induce the appearance of beige cells within WAT.

Activation of peroxisome proliferator-activated receptor-γ (PPARγ) by full agonists, such as thiazolidinediones (TZDs), has been shown to drive browning of WAT [12] by stabilizing PRD1-BF-1-RIZ1 homologous domain-containing protein-16 (PRDM16), a transcriptional coregulator that was previously shown to control the development of genuine brown fat from myoblastic-like precursors [13]. Despite induction of browning in WAT, TZDs do not induce weight loss. This apparent paradox has not been largely explored, but there is evidence indicating that rosiglitazone (RSG) reduces norepinephrine content and turnover rates in BAT and WAT, in addition to decreasing thyroid hormone action [14]. In fact, TZDs induce weight gain and their clinical use has been offset by a number of adverse events, including fluid retention, bone loss, increased risk of cardiovascular events (with RSG) and possibly increased risk of bladder cancer (with pioglitazone), as has been thoroughly reviewed elsewhere [15]. Partial PPARγ agonists, on the other hand, retain the insulin-sensitizing activity of full agonists while not inducing weight gain, fluid retention and bone loss [15]. Nevertheless, some of these partial non-TZD partial PPARγ agonists, such as MRL-24, nTZDpa, Mbx-102 and BVT.13, were not able to induce browning of WAT [13].

We have previously described a partial selective PPARγ agonist, the TZD-derivative GQ-16, that is less adipogenic in cell culture and *in vivo*, and reverses high fat diet-induced insulin resistance and glucose intolerance similarly to RSG in mice but without inducing weight gain [16]. However, its effects on BAT and on browning of WAT were not addressed. In the present study, we explore the effects of GQ-16 on adipose tissue and show that its favourable metabolic effects are accompanied by reduced epididymal fat mass and cell size, morphological signs of increased brown adipose tissue activity (reduced mass, and intracellular lipid) and reduced liver triglyceride content. In addition, GQ-16 treatment induced the expression of thermogenesis-related genes and increased UCP-1 protein expression in BAT and epididymal white adipose, and also resulted in a trend towards increased expression of beige-selective genes in inguinal white adipose tissue of Swiss male mice with diet-induced obesity.

## Material and Methods

### Animals

Three-week-old male Swiss outbred mice, purchased from the Centre for the Development of Experimental Models for Medicine and Biology, Federal University of São Paulo. All mice were housed in plastic mini-isolators in ventilated racks (Alesco, São Paulo, Brazil) in groups of 4 mice per cage in a temperature-controlled room (25°C) with a 12-h light/dark cycle (darkness between 6 pm and 6 am) and had free access to food and water. All procedures were conducted in strict accordance with the recommendations in the *National Institutes of Health’s Guide for the Health and Use of Laboratory Animals* (Institute of Laboratory Animal Resources, 1996) and were approved by the Institutional Animal Use Committee of the University of Brasilia (Permit Number: 27455/2011). All efforts were made to minimize suffering.

### Diet-induced obesity mouse model and experimental design

Mice were fed a control diet (10% kcal as fat; D12450B, Research Diets Inc., New Brunswick, US) or high-fat diet (HFD, 60% kcal as fat; D12492, Research Diets Inc., New Brunswick, US) since weaning (3 weeks of age) to the age of 18 weeks to promote obesity and hyperglycemia. The composition of two diets are shown in S1 Table. At this time, they were randomly assigned into 4 groups with 4 mice each to receive vehicle (0.25% [v/v] Tween-20 diluted in saline, Sigma-Aldrich, St. Louis, US), RSG (Cayman Chemical, Ann Arbor, US; 4 mg/kg/d) or GQ-16 (40 mg/kg/d) by gavage daily for two weeks. We decided to treat mice with 40 mg/kg/d of GQ-16 because previous experiments in our laboratory using 5, 10, 20 and 40 mg/kg/d of GQ-16 demonstrated reduction of fasting blood glucose levels and HFD-induced weight gain in a dose-dependent manner (data not shown for treatment with 5, 10 and 20 mg/kg/d of GQ-16). GQ-16 [(*5Z*)-5-(5-bromo-2-methoxy-benzylidene)-3-(4-methyl-benzyl)-thiazolidine-2,4-dione; CAS 870554-67-9] was synthesized in a manner similar to that previously described [17]. At the end of treatment, all mice were euthanized by decapitation between 9 to 10 am.

Body weight and food and water intake were measured weekly from 3 to 16 weeks of age, and daily during drug treatment (16 to 18 weeks of age). During the latter period, weight gain and energy intake were calculated. Energy intake was calculated by measuring food consumption, and data were presented as the food weight multiplied by its energy content and number of days. Metabolic efficiency was calculated as the body weight gain divided by the energy intake over the period of time of drug treatment (14 days).

At 16 and 18 weeks of age, mice were fasted overnight and blood samples from the dorsal tail vein were collected for blood glucose measurement by using the Accu-chek Performa blood glucose monitor (Roche, US). After animals were euthanized by decapitation, trunk blood was collected, centrifuged (4000 *g* for 15 minutes at 4°C) and serum was stored at -80°C for measurement aspartate aminotransferase (AST), alanine aminotransferase (ALT), high-density lipoprotein cholesterol (HDL-c) and triglyceride concentrations. Two different WAT depots (inguinal and epididymal), one BAT depot (interscapular BAT) and heart were dissected and weighted. Samples of each depot were processed for histological and immunohistochemistry analysis or snap-frozen on liquid nitrogen and stored at -80°C for mRNA expression analysis. Liver samples were also removed for histological analysis and determination of hepatic triglyceride content.

### Serum Biochemical Analysis

Serum AST, ALT, HDL-c, and triglyceride concentrations were analyzed using enzyme assay kits (#OSR6009, OSR6107, OSR6116, OSR60118, respectively), according to the manufacturer´s instructions, on the Beckman Coulter AU680 Chemistry System Automatic Analyzer (Beckman Coulter, Inc., Brea, US).

### Histology

Small fragments from interscapular BAT, inguinal WAT, epididymal WAT, and liver specimens were removed and dissected. During dissections, a standard procedure was maintained to minimize any variance in tissue collection between different mice. Specimens for microscopy were fixed in 4% paraformaldehyde for 24 hours, dehydrated, embedded into a paraffin block, cut into 5 μm-thick sections, stained with hematoxylin and eosin by standard procedures for histological analysis or stained for UCP-1 protein by immunohistochemistry.

The images of each tissue slice were captured with a digital camera mounted on a light-microscope (Axio imager A1, Zeiss Inc., Jena, Germany) with a magnification of 10x for interscapular BAT, 10x for WAT, and 10x for the liver.

A morphometric study of the adipocytes was performed using the Image Pro Plus Software (Media Cybernetics, Rockville, US). For the determination of mean adipocyte diameter, seven representative images were extracted from the whole slide using a Nikon Eclipse 50i microscope with a high-definition color camera DS-Fil-U2 (Nikon Instruments Inc., Melville, US) and image analysis software NIS Elements (Nikon Instruments Inc., Melville, US) at 40x magnification (Nikon Instruments Inc., Melville, US). Individual adipocyte diameter measurements were carried out in a blind fashion using the image analysis software Image-Pro Plus. Approximately 10 non-overlapping adipocytes per image were measured. To determine adipocyte diameter, an image with a known linear scale bar was used and a line was drawn over the image to measure the greater distance between two points in the adipocyte cell membrane. Measurements were obtained from animals in each experimental group and to ensure the accuracy of measurements images from each animal were analyzed by two different investigators.

### Immunohistochemistry

For UCP-1 staining, adipose tissue slides were deparaffinized in xylene, hydrated in 100% and 70% ethanol, and rinsed in water before heat-mediated antigen retrieval in 10 mM sodium citrate buffer (pH 6.0) for 20 minutes, 97°C. UCP-1 imunohistochemistry staining was measured using R.T.U. Vectastain Universal Quick Kit (PK-7800, Vector Laboratories, Inc., CA, US) with minor modifications. Slides were blocked with 2.5% normal horse serum (Vector, S-2012), followed by incubation with rabbit polyclonal UCP-1 primary antibody diluted 1:100 (sc-6528, Santa Cruz Biotechology Inc., Santa Cruz, CA, US) overnight at 4°C. After incubation the slides were washed in PBS for 5 minutes and then incubated with prediluted biotinylated pan-specific universal secondary antibody for 10 minutes (Vector) followed by incubation with ready-to-use streptavidin/peroxidase complex reagent for 5 minutes. After washing the slides in PBS for 5 minutes, they were incubated with substrate solution (Vector NovaRED substrate kit) for 1 minute and then counterstained with Harris hematoxylin. NovaRED substrate produces a red stain in UCP-1 protein and hematoxylin stains nuclei blue-violet. Whole-slide digital images were collected at 20× magnification for iBAT and 10x for WAT with an Aperio Scan Scope slide scanner (Aperio, Vista, CA, US).

### Hepatic triglyceride content quantification

Hepatic triglyceride content was quantified as described previously [18] with slight modifications. Briefly, frozen liver tissues (~100 mg) were homogenized in 2:1:0.8 chloroform:methanol:PBS mixture and then centrifuged. The organic layer was removed and triglyceride content was measured by the enzymatic method using a commercial kit (#OSR60118, Beckman Coulter, Inc., Brea, US).

### RNA isolation, cDNA synthesis, and Quantitative Real-Time PCR Analysis

Total ribonucleic acid (RNA) from tissues was isolated using TRIzol reagent (Invitrogen, California, US) and the chloroform-isopropanol extraction method, following the manufacturer’s protocol. RNA concentration and quality from all samples were checked by a spectrophotometer (NanoVue Plus, GE Healthcare Life Sciences, Buckinghamshire, UK). RNA integrity was checked by verifying the staining intensity of the 28S and 18S rRNA bands after agarose gel electrophoresis. RNA was treated with RNase-free DNase I (Sigma-Aldrich, St. Louis, US) to remove possible contaminating genomic DNA, and 5 ng of total RNA were reverse transcribed (RT). Quantitative real time PCR (qPCR) was carried out using Power SYBR^**®**^ Green RNA-to-CT^™^ 1-Step kit (Applied Biosystems, Foster City, US), along with (5 pmol/μL) primers, and was performed in an AB7500 PCR machine (Applied Biosystems, Foster City, US). Relative mRNA expression was calculated by the comparative threshold cycle (*C*t) method, according to the formula 2^-ΔΔ*C*t^ [19], using *Gapdh* as a reference gene. Primer sequences are shown in S2 Table.

### Statistical analysis

Data were analyzed using the GraphPad Prism 5.0 statistical package. All results are presented as mean ± SEM. Analysis were performed using two-tailed unpaired Student's t-test or one-way ANOVA followed by Newman-Keuls post hoc test for comparing the means of two or multiple groups, respectively. Relative mRNA expression was analyzed using the non-parametric Kruskal-Wallis test followed by Dunn´s test post hoc test. Statistical significance was noted when *p* < 0.05.

## Results

### GQ-16 reduces weight gain, blood glucose levels and visceral adiposity

At weaning, mice were divided into two groups and body weight was similar between them (21.71±3.4, n = 4; *vs* 21.66±0.9, n = 12, *p* > 0.05—two-tailed unpaired Student's t-test). At the age of 16 weeks, after 13 weeks of HFD, mice exhibited a trend towards increased body weight (48.4±5.2 *vs* 57.9±3.7, *p* = 0.05—two-tailed unpaired Student's t-test), significantly higher body weight gain (26.7±2.2 *vs* 37.4±3.7, *p* < 0.05—two-tailed unpaired Student's t-test) and mean fasting blood glucose levels (71.3±2.4 *vs* 116.4±5.4, *p* < 0.05—two-tailed unpaired Student's t-test) compared with mice on a control diet.

**Fig 1:**
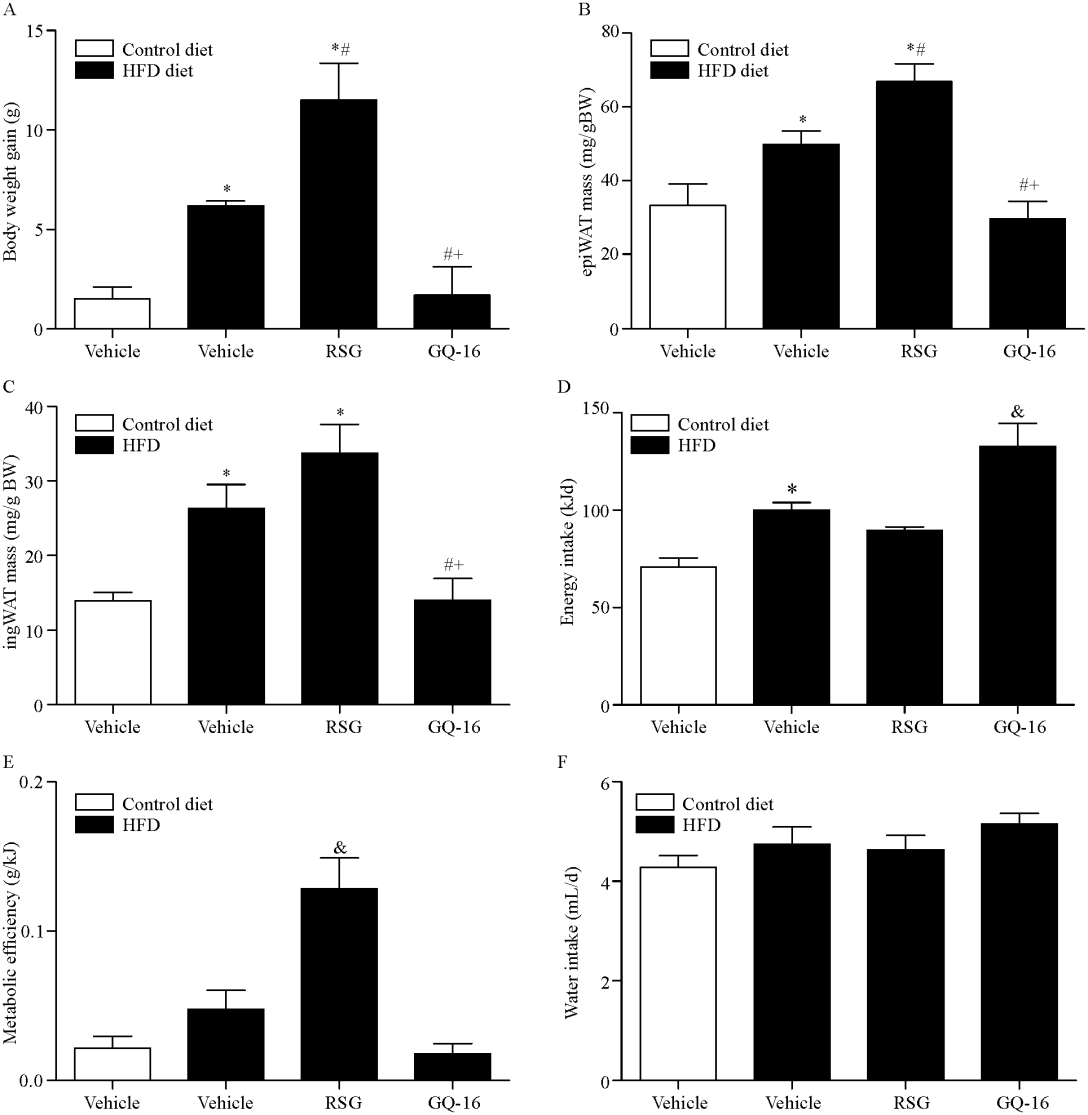
GQ-16 treatment reduces diet-induced weight gain and visceral adipose tissue mass despite increasing energy intake. (**A**) Body weight gain, (**B**) epididymal (epiWAT) fat pad mass, (**C**) inguinal (ingWAT) fat pad mass, (**D**) energy intake, (**E**) metabolic efficiency and (**F**) daily water intake after treatment with vehicle, rosiglitazone (RSG, 4 mg/kg/d), or QG-16 (40 m/kg/d) for two weeks. Visceral adiposity was expressed as the ratio of visceral fat weight to body weight. Subcutaneous adiposity was expressed as the ratio of subcutaneous fat weight to body weight. Data are presented as mean ± SEM. Statistical analysis was done using ANOVA followed by Newman-Keuls post hoc test. * *p* < 0.05 *vs* control diet group that received vehicle, # *p* < 0.05 *vs* HFD group that received vehicle, + *p* < 0.05 *vs* HFD that received rosiglitazone, & *p* < 0.05 *vs* all other groups. n = 4 animals per group.

After two weeks of RSG treatment, at the age of 18 weeks, mice exhibited increased body weight (Fig 1A and S3 Table) and reduced fasting blood glucose levels (Table 1) compared with vehicle-treated mice on HFD. GQ-16 treatment for the same period reduced both weight gain (Fig 1A and S3 Table) and fasting blood glucose levels (Table 1) to values comparable to those of mice fed a control diet (Fig 1A, Table 1 and S3 Table).

**Table 1:** Effects of two week-GQ-16 treatment on serum blood glucose, lipid profile and transaminases in male Swiss mice.

	Control diet	HFD + vehicle	HFD + RSG	HFD + GQ-16
Glucose (mg/dL)	69.8±3.2	121.0±7.4*	100.0±7.9^#^	69.0±5.1^#^^,^^+^
HDL-c (mg/dL)	57.0±2.8	52.0±3.1	54.5±4.0	64.5±4.8
Triglycerides (mg/dL)	138.7±13.7	112.0±12.4	101.5±4.9	98.5±7.9
AST (U/L)	164.5±27.3	348.0±68.4	263.0±29.5	279.7±88.1
ALT (U/L)	42.8±12.9	80.0±21.3	136.3±49.0	50.8±19.5

Data are given as mean ± SEM. Statistical analysis was done using ANOVA followed by Newman-Keuls post hoc test.

* *p* < 0.05 *vs* control diet that received vehicle;

^#^ *p* < 0.05 *vs* HFD group that received vehicle;

^+^ *p* < 0.05 *vs* HFD that received RSG.

n = 4 animals per group. ALT, alanine aminotransferase; AST, aspartate aminotransferase; HDL-c, high-density lipoprotein-cholesterol; HFD, high fat diet; RSG, rosiglitazone.

Reduced weight gain in mice treated with GQ-16 was associated with reduced visceral and subcutaneous adiposity, as indicated by reduced epididymal and inguinal fat mass (Fig 1B and 1C, S3 Table) compared with vehicle-treated mice on HFD. RSG, on the other hand, increased the mass of both fat depots (Fig 1B and 1C, S3 Table). Cardiac mass not changed in response to treatment with GQ-16 or RSG (S3 Table).

Neither treatment modified triglyceride or HDL-c serum levels, although there was a slight trend towards increased HDL-c levels in response to GQ-16 treatment (Table 1). Surprisingly, GQ-16 treatment increased energy intake despite reducing weight gain (Fig 1D), and this resulted in lower metabolic efficiency (Fig 1E). Neither treatment induced changes in water intake (Fig 1F) or circulating aspartate and alanine aminotransferase levels (Table 1).

### GQ-16 reduces hepatic triglyceride accumulation

**Fig 2:**
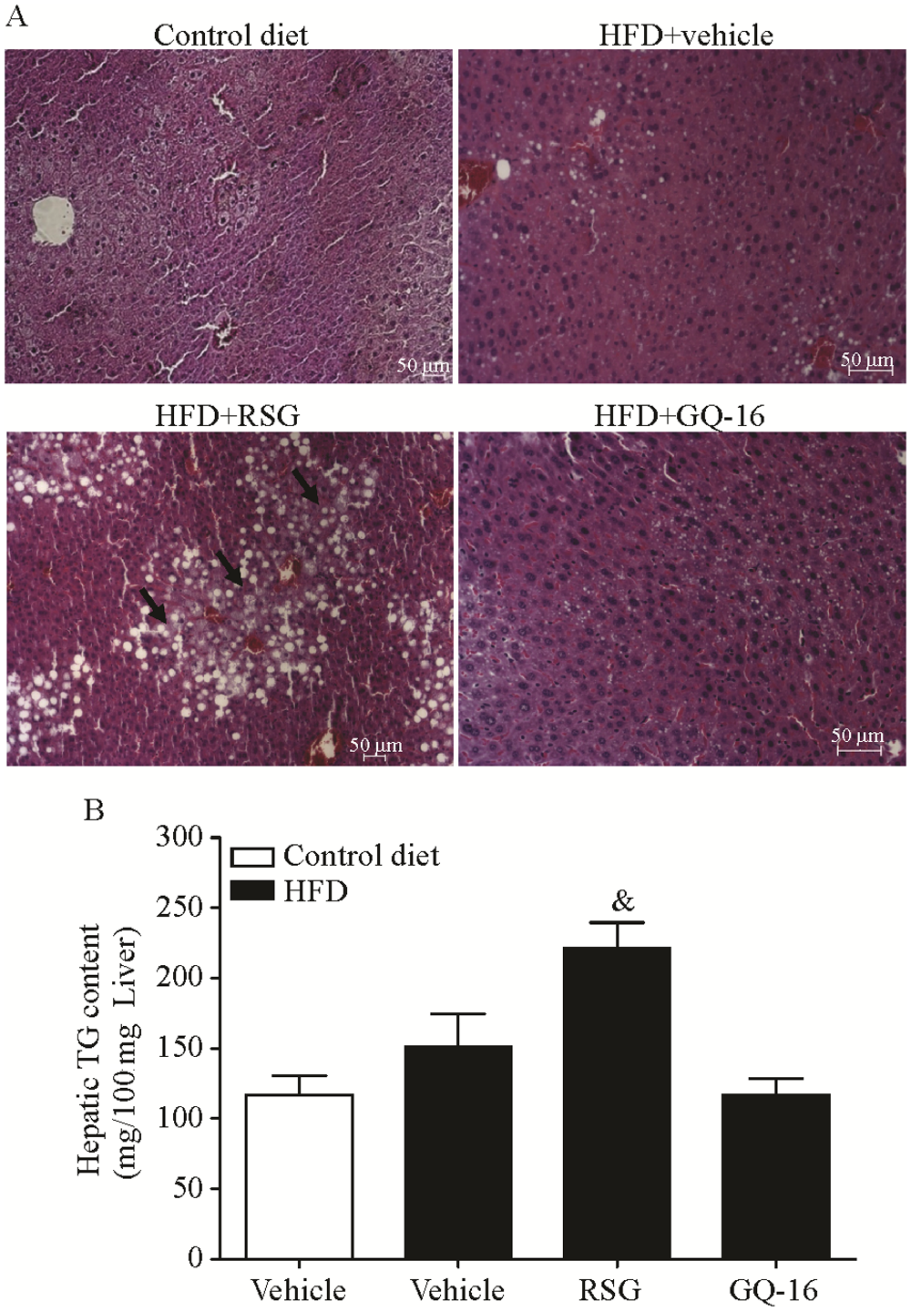
GQ-16 treatment reduces hepatic triglyceride accumulation. **(A)** Sections of liver specimens stained with hematoxylin-eosin (representative pictures with magnification x10; scale bar, 50 μm) and (**B**) liver triglyceride content after treatment with vehicle, rosiglitazone (RSG, 4 mg/kg/d), or QG-16 (40 m/kg/d) for two weeks. Data are presented as mean ± SEM. Statistical analysis was done using ANOVA followed by Newman-Keuls post hoc test. & *p* < 0.05 *vs* all other groups. n = 4 animals per group.

Liver histological analysis from HFD-fed mice showed slight lipid accumulation when compared to mice fed a control diet, and this effect was exacerbated in response to RSG treatment (Fig 2A). RSG treatment increased (by 46%) hepatic triglyceride content whereas GQ-16-treated mice exhibited a slight trend towards decreased (by 24%) hepatic triglyceride content when compared to vehicle-treated mice fed a HFD (Fig 2B).

### GQ-16 effects on adipocyte morphology suggest activation of brown adipose tissue and browning of white adipose tissue

**Fig 3:**
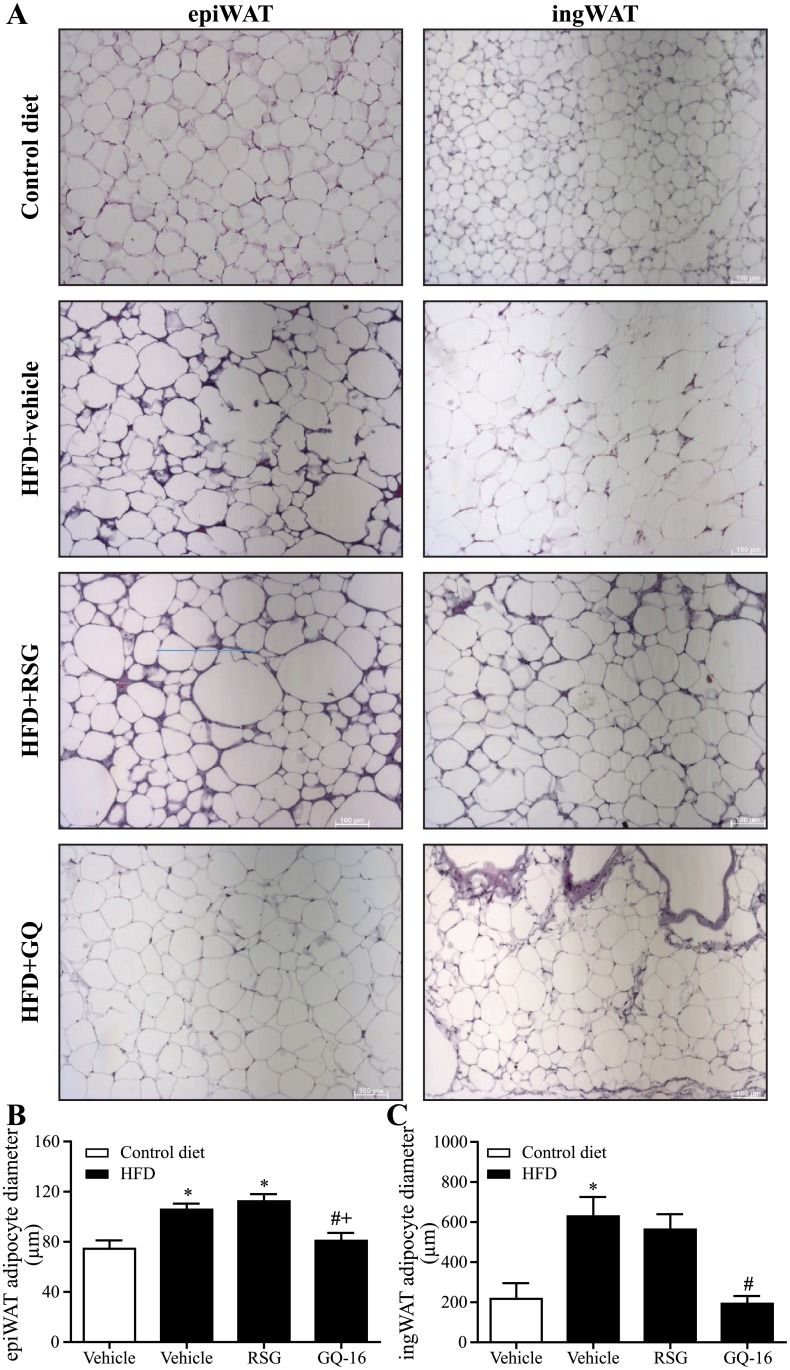
GQ-16 treatment reduces white adipocyte size. (**A**) Sections of visceral white adipose tissue (epiWAT, left panel) and subcutaneous white adipose tissue (ingWAT, right panel) stained with hematoxylin-eosin (images are shown at 10x magnification. Scale bar, 100 μm) and diameter of adipocytes in (**B**) epiWAT and (**C**) ingWAT obtained from mice after treatment with vehicle, rosiglitazone (RSG, 4 mg/kg/d), or QG-16 (40 m/kg/d) for two weeks. Data are given as mean ± SEM. Statistical analysis was done using ANOVA followed by Newman-Keuls post hoc test. * *p* < 0.05 *vs* control diet group that received vehicle, # *p* < 0.05 *vs* HFD group that received vehicle, + *p* < 0.05 *vs* HFD that received rosiglitazone. n = ~ 4 animals per group.

In order to gain insights into the favorable metabolic effects of GQ-16 treatment, particularly reduced weight gain in response to HFD in the setting of increased energy intake, we investigated WAT and BAT morphology. Histological analysis suggested that RSG treatment enhanced adipocyte hypertrophy in response to HFD in both inguinal and epididymal WAT depots, whereas GQ-16 treatment attenuated this effect (Fig 3A). The average adipocyte diameter of HFD-fed mice treated with GQ-16 was smaller than that of HFD-fed mice treated with vehicle or RSG and similar to vehicle-treated mice fed a control diet (Fig 3B and 3C).

**Fig 4:**
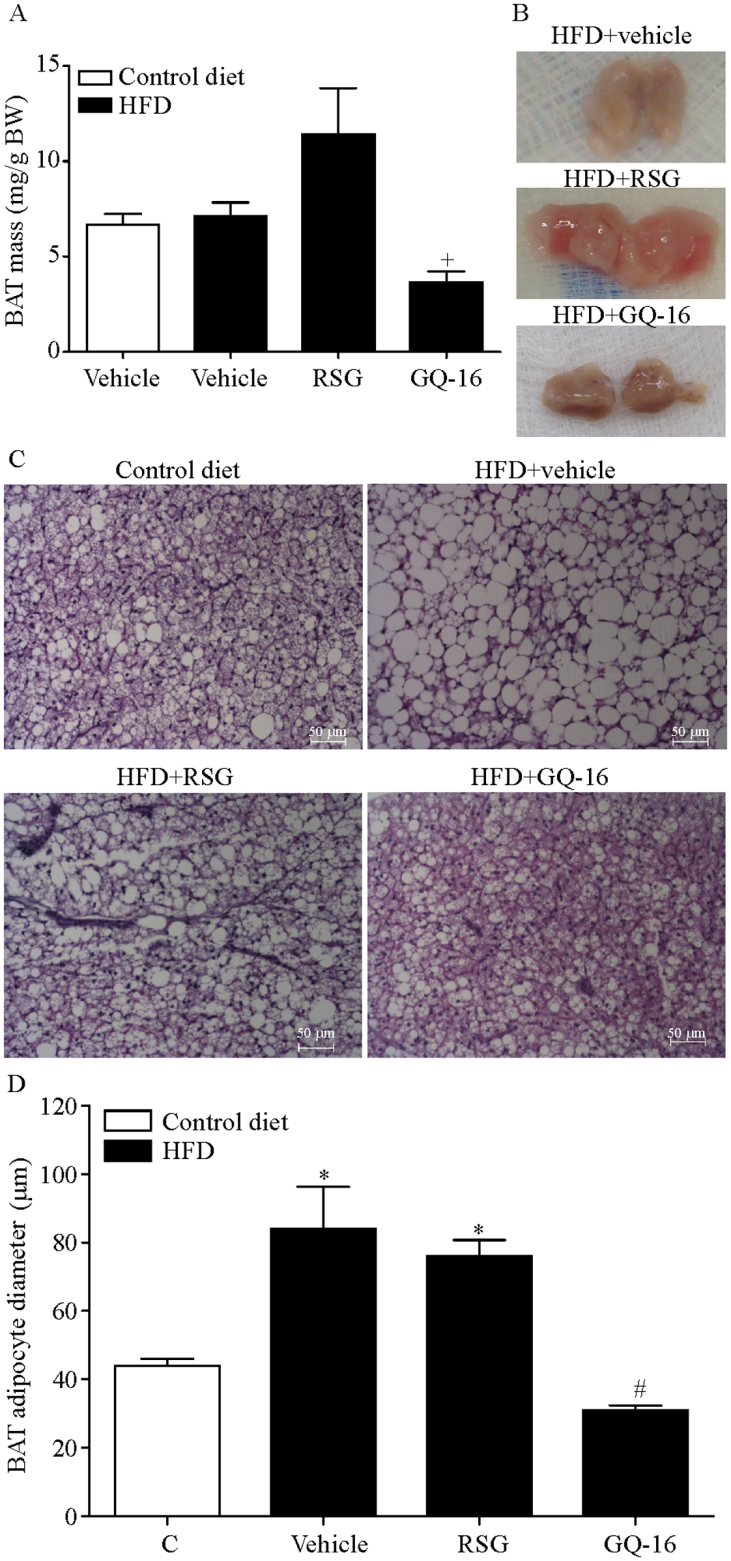
GQ-16 reduces brown adipose tissue mass and adipocyte diameter. (**A**) Interscapular brown adipose tissue (iBAT) fat pad mass, (**B**) gross appearance of iBAT, (**C**) sections of iBAT stained with hematoxylin-eosin (images are shown at 10x magnification; scale bar, 50 μm) and (**D**) mean diameter of brown adipocytes obtained from mice after treatment with vehicle, rosiglitazone (RSG, 4 mg/kg/d), or QG-16 (40 m/kg/d) for two weeks. Data are given as mean ± SEM. Statistical analysis was done using ANOVA followed by Newman-Keuls post hoc test. + *p* < 0.05 *vs* HFD that received rosiglitazone; * *p* < 0.05 *vs* control diet group that received vehicle, # *p* < 0.05 *vs* HFD that received RSG or vehicle.

Moreover, oversized interscapular BAT was apparent in RSG-treated mice on a HFD. On the other hand, this BAT depot was darker and showed a trend towards decreased (by 49.7%) mass in response to GQ-16 treatment (Fig 4A and 4B). Histological analysis showed that interscapular BAT depot from HFD-fed mice treated with vehicle or RSG contained big lipid droplets. On the contrary, in HFD-fed mice treated with GQ-16 interscapular BAT had small multilocular adipocytes, similarly to control diet-fed mice (Fig 4C), as indicated by the smaller size of the intracellular lipid droplets. The average adipocyte diameter of HFD-fed mice treated with GQ-16 was similar to vehicle-treated mice fed a control diet (Fig 4D).

### GQ-16 treatment induces the expression of thermogenesis-related genes in interscapular brown adipose tissue and epididymal white adipose tissue

**Fig 5:**
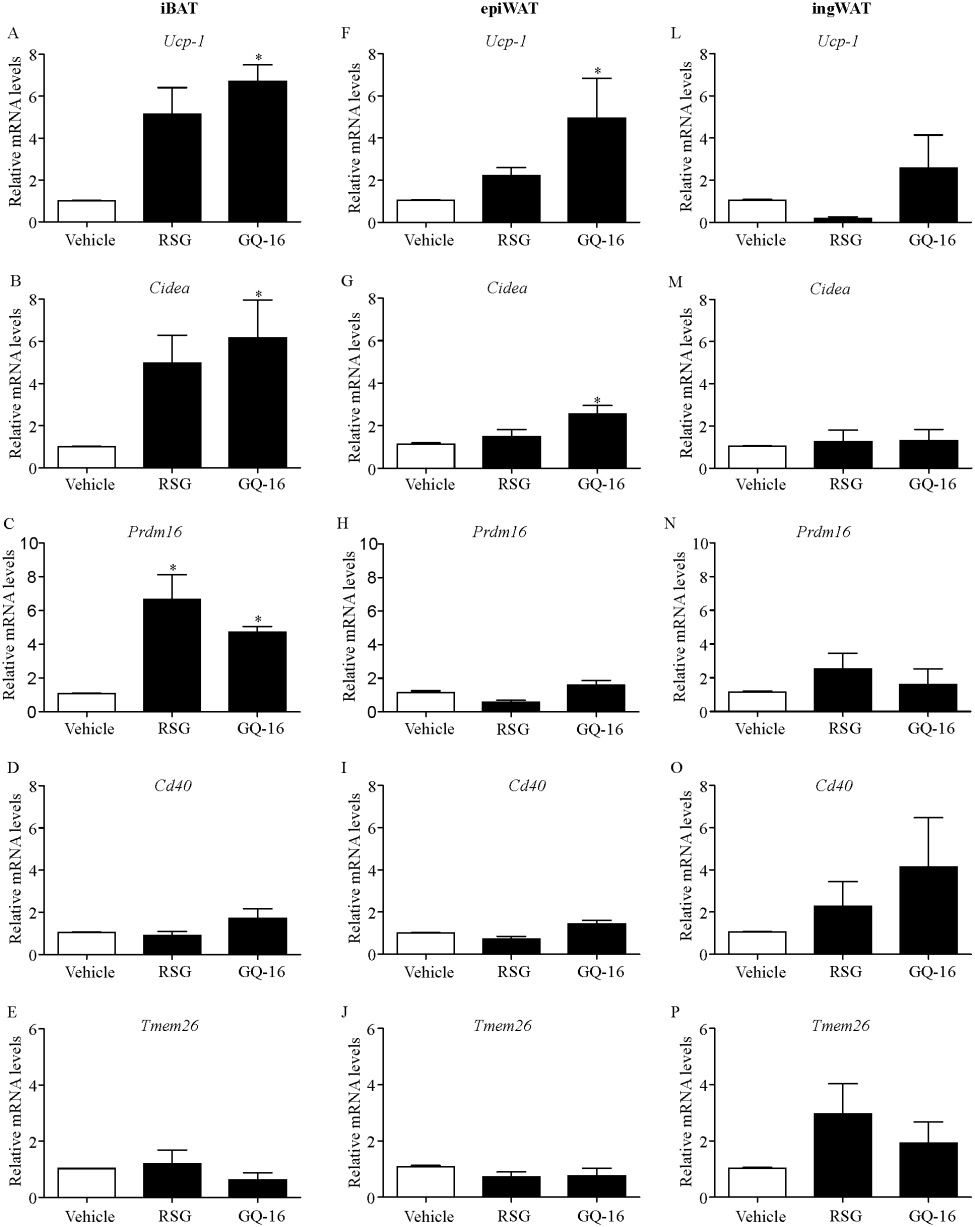
GQ-16 treatment induces the expression of thermogenesis-related genes in brown and white adipose tissue. Relative mRNA expression for (**A, F, L**) *Ucp-1*, (**B, G, M**) *Cidea*, (**C, H, N**) *Prdm16*, (**D, I, O**) *Cd40*, (**E, J, P**) *Tmem26* in iBAT, epiWAT and ingWAT in mice treated with vehicle, rosiglitazone (RSG, 4 mg/kg/d), or QG-16 (40 m/kg/d) for two weeks. Gene expression in the iBAT, epiWAT and ingWAT was determined using qRT-PCR and normalized to levels of *Gapdh*. Data are given as mean ± SEM. Statistical analysis was done using non-parametric Kruskal-Wallis test followed by Dunn´s test post hoc. * *p* < 0.05 *vs* HFD group that received vehicle. n = 4 animals per group. iBAT, interscapular brown adipose tissue; ingWAT inguinal white adipose tissue; epiWAT epididymal white adipose tissue.

Reduced weight gain and reduced visceral adiposity induced by GQ-16 treatment in the setting of increased energy intake, in addition to the findings on adipocyte morphology, prompted us to investigate the expression of thermogenesis-related genes in brown (interscapular) and white (epididymal and inguinal) adipose tissue depots. In interscapular brown adipose tissue, GQ-16 treatment increased the expression of *Ucp-1*, *Cidea* and *Prdm16* compared with vehicle-treated mice on HFD (Fig 5A–5C). In this same fat depot, RSG treatment induced a non-significant increase in the relative expression levels of *Ucp-1* and *Cidea*, and a significant increase in *Prdm16* levels (Fig 5A–5C). In epididymal WAT, GQ-16 increased the expression of *Ucp-1*, *Cidea* but not *Prdm16* compared with vehicle-treated mice on HFD (Fig 5F–5H), whereas RSG treatment did not change the relative expression levels of these transcripts (Fig 5F–5H). There were no changes in the expression of thermogenesis-related genes in the inguinal WAT in response to either RSG or GQ-16 treatment (Fig 5L–5N).

The expression of beige adipocyte-selective genes *Cd40* and *Tmem26* was not changed in response to treatment with GQ-16 in interscapular BAT and both WAT depot analyzed (epididymal and inguinal) (Fig 5D, 5E, 5I, 5J, 5O and 5P). There was a slight trend towards increased mRNA levels of *Cd40* and *Tmem26* in the inguinal WAT of GQ-16 and RSG-treated mice compared with vehicle-treated mice on HFD (Fig 5O and 5P). The levels of these transcripts did not change in response to GQ-16 or RSG treatment in epididymal WAT or interscapular BAT (Fig 5D, 5E, 5I and 5J).

### GQ-16 treatment induces the expression of UCP-1 in interscapular brown adipose tissue and white adipose tissue

**Fig 6:**
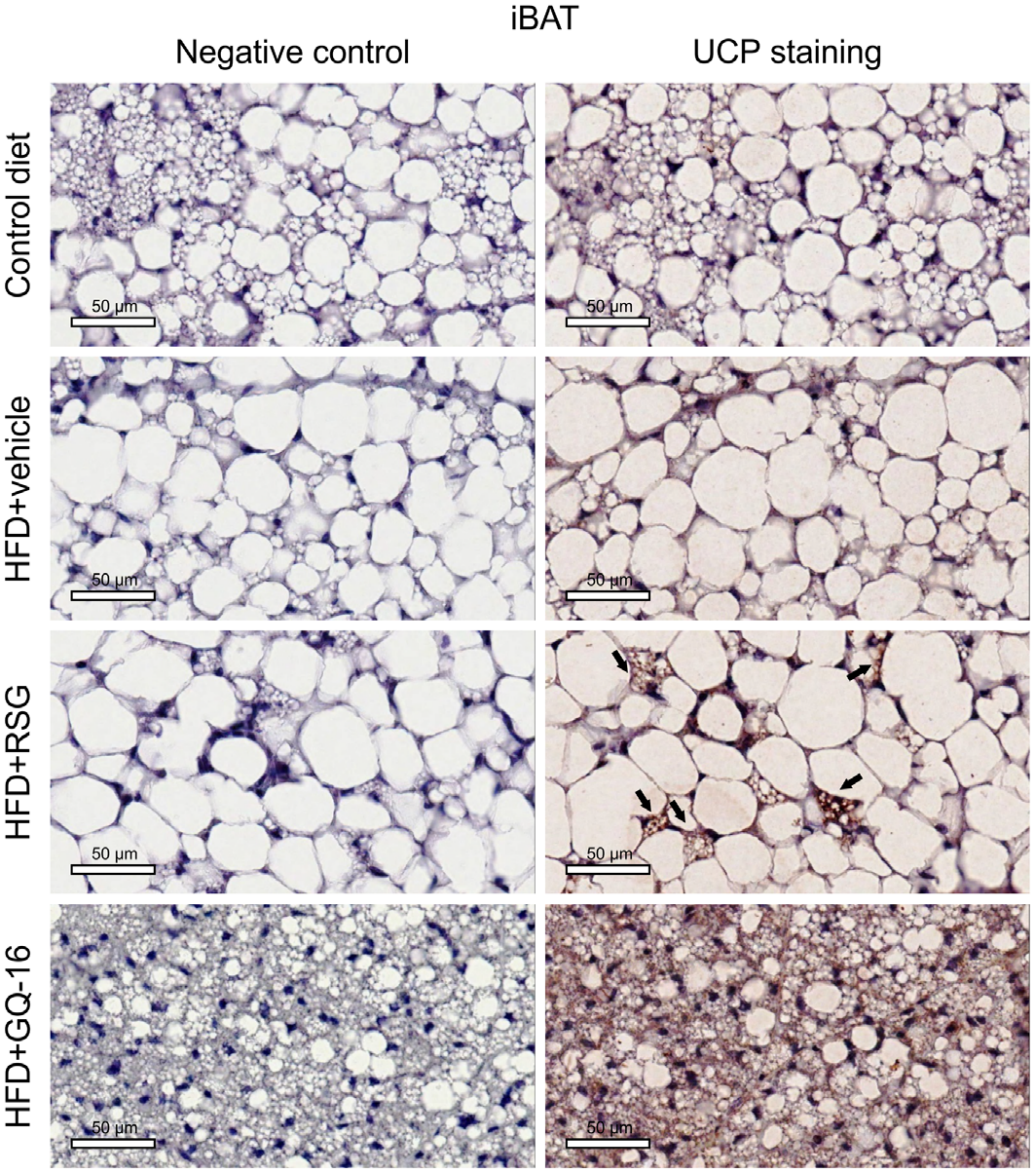
GQ-16 treatment increases UCP-1 expression in interscapular brown adipose tissue. UCP-1 immunostaining in sections of interscapular brown adipose tissue (iBAT) obtained from mice after treatment with vehicle, rosiglitazone (RSG, 4 mg/kg/d), or QG-16 (40 mg/kg/d) for two weeks. Representative Images (three total images per group) are shown at 20x magnification, scale bar, 50 μm. Slides were stained with NovaRED substrate that produces a red-stain in UCP-1 protein and they were counterstained with hematoxylin that results in blue-violet staining of nuclei.

**Fig 7:**
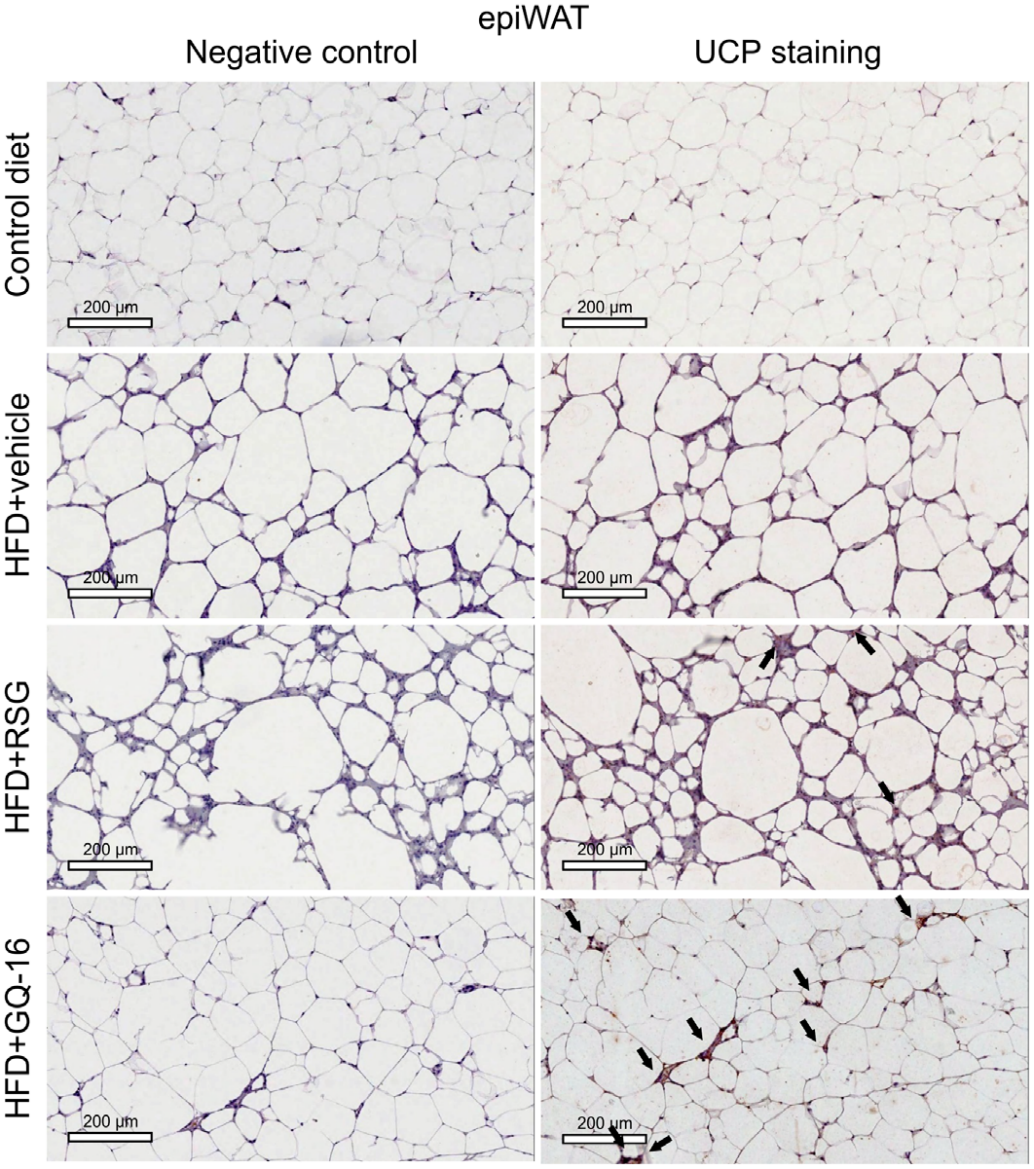
GQ-16 treatment induces UCP-1 expression in visceral white adipose tissue. UCP-1 immunostaining in sections of epididymal white adipose tissue (epiWAT) obtained from mice after treatment with vehicle, rosiglitazone (RSG, 4 mg/kg/d), or QG-16 (40 mg/kg/d) for two weeks. Representative Images (three total images per group) are shown at 10x magnification, scale bar, 50 μm. Slides were stained with NovaRED substrate that produces a red-stain in UCP-1 protein and they were counterstained with hematoxylin that results in blue-violet staining of nuclei.

**Fig 8:**
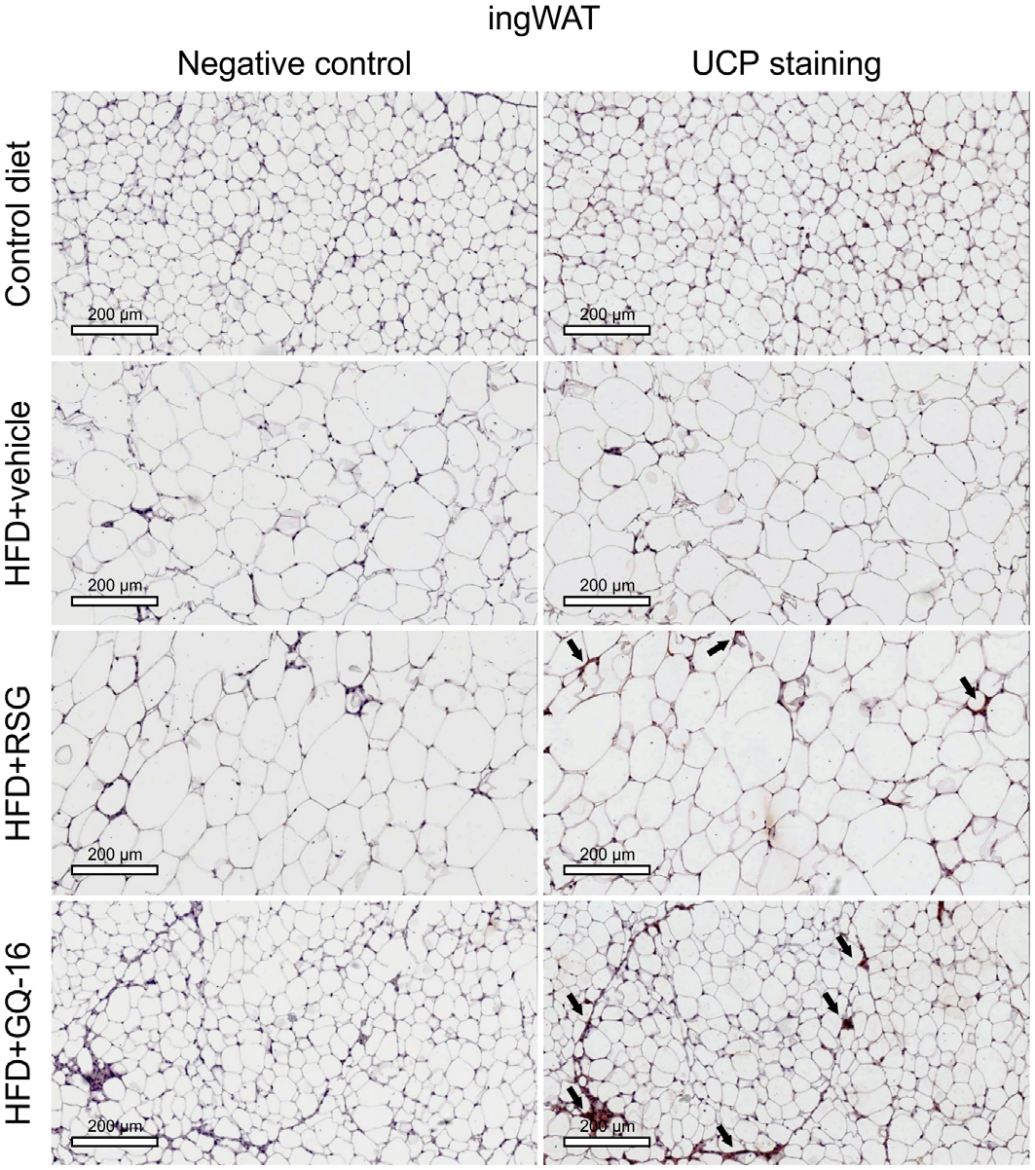
GQ-16 treatment induces UCP-1 expression in subcutaneous white adipose tissue. UCP-1 immunostaining in sections of inguinal white adipose tissue (ingWAT) obtained from mice after treatment with vehicle, rosiglitazone (RSG, 4 mg/kg/d), or QG-16 (40 mg/kg/d) for two weeks. Representative Images (three total images per group) are shown at 10x magnification, scale bar, 50 μm. Slides were stained with NovaRED substrate that produces a red-stain in UCP-1 protein and they were counterstained with hematoxylin that results in blue-violet staining of nuclei.

Immunohistochemistry analysis showed that the interscapular BAT depot from HFD-fed mice treated with GQ-16 contained more UCP1-positive cells than that from HFD-fed mice treated with vehicle or RSG (Fig 6). Furthermore, epididymal WAT and inguinal WAT in QG-16- and RSG-treated mice exhibited more UCP1-positive cells when compared with HFD-fed mice treated with vehicle (Figs 7 and 8).

## Discussion

The interest for PPARγ-based therapies has been renewed over the last few years by increased understanding of the mechanisms of action of TZDs and the PPARγ-regulated molecular pathways leading to beneficial metabolic effects. It was recently shown that the insulin-sensitizing activity of PPARγ agonists might not be solely related to their classical agonism, but also to their ability of blocking cyclin-dependent kinase 5-mediated phosphorylation of PPARγ at Ser273 [20, 21]. Full PPARγ activation by TZDs also induces browning of WAT, whereas it was shown that partial agonists were devoid of this effect [13]. In the present study, we showed that the TZD-derivative GQ-16, a previously described partial PPARγ agonist [16], induces a gene expression pattern suggestive of brown-like or beige adipocyte emergence within the epididymal white adipose tissue depot male Swiss mice with obesity and hyperglycemia induced by HFD.

Treatment of obese and hyperglycemic male Swiss mice with GQ-16 reduced fasting blood glucose to levels comparable to those of lean mice. In contrast to full PPARγ agonists, which induce weight gain [15] and, in rodents, induce liver steatosis [22–25], GQ-16 treatment decreased HFD-induced weight gain and visceral WAT mass, in addition to decreasing triglyceride liver content to values similar to those seen in lean mice on a control diet. Reduced weight gain occurred in the setting of increased energy intake, and this suggested energy expenditure might have been higher in GQ-16 treated animals. Since GQ-16 is a selective PPARγ ligand [16] and PPARγ is abundantly expressed in white adipose tissue [26], this finding prompted us to investigate whether GQ-16 induced browning of WAT. Although we did not assess energy expenditure, we found that treatment with GQ-16 reduced metabolic efficiency, increased mRNA levels of *Ucp-1* and *Cidea* in epididymal fat, but not in the subcutaneous (inguinal) WAT. This was accompanied by decreased epididymal fat mass and epididymal adipocyte size, decreased lipid content, and contained more UCP1-positive cells. These findings may suggest increased lipolysis and uncoupled respiration at this site, which may have in turn contributed to reduced weight gain. We cannot exclude that the possible increase in energy expenditure might have been also mediated by BAT activity in GQ-16 treated mice. This group exhibited a darker appearance of BAT and decreased brown adipocyte lipid droplet size, in addition to increased amount of UCP1-positve cells and increased expression of *Ucp-1* mRNA levels, which are features similar to those of activated BAT in cold-exposed mice [1, 27].

The effect of full PPARγ agonists to promote BAT-like features (increased mitochondrial biogenesis and/or increased expression of *Ucp-1*) in WAT has been well established more that a decade ago [12, 28–30]. Recent studies have provided unequivocal evidence that BAT-like cells in WAT comprise a distinct cell type, with a unique cellular origin [5, 31] and gene expression pattern [5]. These studies have also provided insights into the mechanisms underlying browning of WAT upon PPARγ activation. It was shown browning of WAT by full PPARγ agonists preferentially occurs in subcutaneous over visceral white fat depots and is largely due to stabilization and increased levels of *Prdm16* [13], a transcriptional regulator implicated in brown fat [32] and also in beige adipocyte development [33]. The browning effects of PPARγ ligands are associated with increased oxygen consumption only when combined with ß3-adrenergic treatment, both in cell culture [12] and *in vivo* [29]. This is consistent with the observation that TZD treatment *per se* does not increase energy expenditure [34]. In spite of inducing a thermogenic genetic program in WAT, which would be expected to result in increased energy expenditure and possibly weight loss, TZDs are associated with weight gain [15]. On the other hand, some partial PPARγ agonists decrease weight gain in obesity models [35–37], raising the possibility that their effect on weight would be associated with increased browning of WAT, and even increased thermogenesis. However, non-TZD partial agonists such as MRL-24, nTZDpa, Mbx-102 and BVT-13 did not show any effect on the expression of thermogenesis-related genes in WAT [13]. In contrast with these data, our findings indicate that the partial TZD-derived PPARγ agonist GQ-16 increases the expression of *Ucp-1* and *Cidea* preferentially in visceral WAT. The emerging questions are the reasons for the preferential induction of *Ucp-1* and *Cidea* in visceral over subcutaneous WAT and the possibility of WAT browning induced by a partial PPARγ agonist.

Browning of WAT in response to both sympathetic activation [6, 38] and PPARγ activation by ligands [12] has been shown to vary among different mouse strains [6, 38]. It is also possible that the response of the different WAT depots to the browning effects of PPARγ ligands is genetically determined, so that in some mouse strains there may be a preferential browning effect on subcutaneous WAT, whereas in other strains the effect may be most evident in visceral WAT. We found that in male Swiss mice with HFD-induced obesity there was preferential induction of thermogenesis-related genes (*Ucp-1* and *Cidea*) in visceral (epididymal) WAT in response to treatment with the TZD-derivative GQ-16. Studies with different mouse strains have shown that although in most strains the browning effects of PPARγ ligands occur preferentially in subcutaneous WAT [29, 39], induction of thermogenesis-related genes may also be seen in visceral WAT [28] or even in both types of depots [39]. In addition to the preferential increase in the expression of *Ucp-1* in the visceral WAT of male Swiss mice, our data indicated that there was no induction of thermogenesis-related genes by RSG in both inguinal and epididymal fat pads of these mice. This may either suggest that Swiss mice are less prone to the browning effects of RSG, or that only the fat depots not analysed herein, such as the mesenteric, retroperitoneal or anterior subcutaneous, would respond to PPARγ activation in this mouse strain.

We also found a trend towards increased expression of beige-selective genes (*Tmem26* and *Cd40*) in subcutaneous WAT but not in epididymal WAT in response to both RSG and GQ-16, despite the induction of thermogenesis-related genes in epididymal but not subcutaneous WAT. Beige-selective genes were recently described in adipocytes differentiated in culture from the stromal vascular fraction of the inguinal fat depots of 129SVE mice [5]. It is possible that brown-like adipocytes emerging in visceral WAT in response to PPARγ agonists express low levels of these markers, or even that these cells have a different genetic signature.

The other question is how GQ-16, a partial PPARγ agonist, would promote browning of WAT, since other partial agonists have been shown to have no browning effect. The induction of WAT browning upon PPARγ full but not partial agonism suggests that the conformation of PPARγ ligand-binding domain (LBD) bound to a full agonist is necessary to recruit beige adipocytes in WAT [13]. The hallmark of full agonists, such as TZDs, is to bind to PPARγ LBD and stabilize its helix 12 [40], whereas partial agonists typically modify the conformation of helix 3 and the beta-sheet region and minimally affect helix 12 [41]. We have previously shown that GQ-16, as other partial PPARγ agonists, makes no direct contact with helix 12. Despite this, it induces significant stabilization of helix 12, in a mode similar to that of the full agonist RSG, possibly due to a water molecule mediating an indirect contact between GQ-16 and helix 12 that is sufficient to stabilize this helix [16]. A more stable conformation of helix 12 in the PPARγ-GQ-16 complex may help to explain the browning effects of treatment with this partial agonist when compared to other partial agonists. We can also not exclude that the browning effect of both TZDs and GQ-16 is related to their TZD structure, since other partial agonists that have been investigated with respect to browning of WAT (MRL-24, nTZDpa, Mbx-102 and BVT-13) are non TZD-derived.

WAT browning induced by full PPARγ agonists does not translate into increased adaptive thermogenesis and energy expenditure, in keeping with TZD’s effect of inducing increased metabolic efficiency, increased fat mass and weight gain [1]. The absence of thermogenesis in this setting seems to result, at least in part, from reduced sympathetic activity and thyroid hormone action in adipose tissue in response to full PPARγ activation [14]. It is possible that modulation of PPARγ activity by partial agonists may have different effects on energy homeostasis that otherwise decrease metabolic efficiency. This is plausible in the light of our findings on fat mass and adipocyte morphology. Consistently with the effect of full PPARγ agonists to increase BAT [42] and WAT [43] lipogenesis and mass, we found that RSG treatment increased both BAT and WAT mass. On the other hand, interscapular BAT in GQ-16 treated mice had features similar to those seen in activated BAT in cold-exposed animals, such as reduced mass and darker appearance, in addiction to a decrease in lipid vacuoles [1, 27]. WAT also exhibited reduced adipocyte size in response to GQ-16.

The main limitation of our study is the lack of energy expenditure measurement, even though mice treated with GQ-16 displayed reduced weight gain in the setting of increased energy intake, decreased white and brown fat mass in response to HFD and increased expression of thermogenesis-related genes in both white and brown fat. In our previous study [16], we found no change in energy expenditure or food intake of male C57Bl/6J mice with HFD-induced obesity after three-day treatment with 20 mg/kg/d of GQ-16 administered intraperitoneally. Although GQ-16 induced a decrease in white fat mass after seven days of treatment, the lack of both increased energy expenditure and significantly increased *Ucp-1* in WAT may have presumably occurred because we used a different study design. In the current study, we treated mice with GQ-16 by gavage (not intraperitoneally) using a higher dose (40 mg/kg/d *versus* 20 mg/kg/d of GQ-16) and during a longer period (14 days study *versus* 7 days) when compared to our previous study. This is very important since browning of WAT in response to PPARγ activation by agonists requires treatment for at least ten days [12, 13]. Additionally, we can also not exclude the contribution of increased faecal energy loss to decreased weight gain in the setting of increased energy intake. Moreover, we do not have enough data to address a possible role of toxicity related to GQ-16 treatment on our results. However, we believe this may have not been the case since mice appeared grossly healthy, exhibited no signs liver toxicity on histology and no increase in serum liver enzyme levels, and did not show reduced food or water consumption.

## Conclusion

Our current findings indicate that 14-day treatment of obese Swiss male mice with GQ-16 induces decreased weight gain and visceral WAT mass in response to HFD, despite increasing energy consumption. These effects were accompanied by induction of the expression of thermogenesis-related (*Ucp-1* and *Cidea*) and UCP-1 positive cells in epididymal fat depots, suggesting that browning of visceral WAT may have at least in part contributed to weight loss. The importance of beige adipocytes to energy homeostasis has been clearly demonstrated in rodents [7], and the data indicating that the previously described functional BAT depots in adults are largely composed of beige cells [5, 44] provide a rationale for translating insights on beige adipocyte biology from rodents into humans. In this setting, our results strongly support that PPARγ activation by partial agonists, devoid of full agonism-related unfavourable effects, may be a strategy to induce browning of WAT and hence to treat obesity and diabetes.

## Supporting Information

S1 Table Composition of diets used to promote obesity and hyperglycemia. (DOCX)

S2 Table Primer sequences used for real-time PCR. (DOCX)

S3 Table Effects of two week-GQ-16 treatment on body weight, food intake, cardiac mass, epididymal fat mass, inguinal fat mass and brown fat mass in male Swiss mice. (DOCX)

## Abbreviations

ALT: alanine aminotransferase
AST: aspartate aminotransferase
BAT: brown adipose tissue
CIDEA: cell death-inducing DNA fragmentation factor-alpha-like effector A
CD40: cluster of differentiation 40
DIO: diet-induced obesity
HFD: high fat diet
HDL-c: high-density lipoprotein cholesterol
PPAR: peroxisome proliferator-activated receptor
PRDM16: PRD1-BF-1-RIZ1 homologous domain-containing protein-16
RSG: rosiglitazone
TG: triglyceride
Tmem26: transmembrane protein 26
TZD: thiazolidinedione
UCP-1: uncoupling protein 1
WAT: white adipose tissue
