# Supplementary material for: GQ-16, a TZD-Derived Partial PPARγ Agonist, Induces the Expression of Thermogenesis-Related Genes in Brown Fat and Visceral White Fat and Decreases Visceral Adiposity in Obese and Hyperglycemic Mice
Source: PLoS One. 2016 May 3;11(5):e0154310. doi: 10.1371/journal.pone.0154310 (PMC4854408; doi:10.1371/journal.pone.0154310)
Supplement: S1 Table — (DOCX) [file pone.0154310.s001.docx]

**Supporting Information**

**S1 Table.** Composition of diets used to promote obesity and hyperglycemia.

| Product | Control diet (10% kcal% fat) | | HFD (60% kcal% fat) | |
| --- | --- | --- | --- | --- |
|  | (gm %) | (kcal%) | (gm %) | (kcal%) |
| Protein | 19.2 | 20 | 26.2 | 20 |
| Carbohydrate | 67.3 | 70 | 26.3 | 20 |
| Fat | 4.3 | 10 | 34.9 | 60 |
| Total | 3.85 | 100 | 5.24 | 100 |
| Ingredient |  |  |  |  |
| Casein, 30m | 200 | 800 | 200 | 800 |
| L-Cystine | 3 | 12 | 3 | 12 |
| Corn Starch | 315 | 1260 | 0 | 0 |
| Maltodextrin 10 | 35 | 140 | 125 | 500 |
| Sucrose | 350 | 1400 | 68.8 | 275.2 |
| Cellulose | 50 | 0 | 50 | 0 |
| Soybean Oil | 25 | 225 | 25 | 225 |
| Lard | 20 | 180 | 245 | 2205 |
| Mineral Mix S10026 | 10 | 0 | 10 | 0 |
| DiCalcium Phosphate | 13 | 0 | 13 | 0 |
| Calcium Carbonate | 5.5 | 0 | 5.5 | 0 |
| Potassium Citrate | 16.5 | 0 | 16.5 | 0 |
| Vitamin Mix V10001 | 10 | 40 | 10 | 40 |
| Choline Bitartrate | 2 | 0 | 2 | 0 |
| FD&C Dye* | 0.05 | 0 | 0.05 | 0 |
| Total | 1055.05 | 4057 | 773.85 | 4057 |

Control diet contains FD&C blue dye #1 and HFD contains FD&C yellow blue dye #5. HFD, high fat diet.
