# Supplementary material for: GQ-16, a TZD-Derived Partial PPARγ Agonist, Induces the Expression of Thermogenesis-Related Genes in Brown Fat and Visceral White Fat and Decreases Visceral Adiposity in Obese and Hyperglycemic Mice
Source: PLoS One. 2016 May 3;11(5):e0154310. doi: 10.1371/journal.pone.0154310 (PMC4854408; doi:10.1371/journal.pone.0154310)
Supplement: S2 Table — (DOCX) [file pone.0154310.s002.docx]

**Supporting Information**

**S2 Table.** Primer sequences used for real-time PCR.

| Gene | Forward primer | Reverse primer |
| --- | --- | --- |
| *Ucp-1* | ACTGCCACACCTCCAGTCATT | CTTTGCCTCACTCAGGATTGG |
| *Cidea* | TGCTCTTCTGTATCGCCCAGT | GCCGTGTTAAGGAATCTGCTG |
| *Tmem26* | ACCCTGTCATCCCACAGAG | TGTTTGGTGGAGTCCTAAGGTC |
| *Cd40* | TTGTTGACAGCGGTCCATCTA | CCATCGTGGAGGTACTGTTTG |
| *Prdm16* | CAGCACGGTGAAGCCATTC | GCGTGCATCCGCTTGTG |
| *Gapdh* | AAGGGCTCATGACCACAGTC | CAGGGATGATGTTCTGGGCA |
