# Supplementary material for: GQ-16, a TZD-Derived Partial PPARγ Agonist, Induces the Expression of Thermogenesis-Related Genes in Brown Fat and Visceral White Fat and Decreases Visceral Adiposity in Obese and Hyperglycemic Mice
Source: PLoS One. 2016 May 3;11(5):e0154310. doi: 10.1371/journal.pone.0154310 (PMC4854408; doi:10.1371/journal.pone.0154310)
Supplement: S3 Table — (DOCX) [file pone.0154310.s003.docx]

**Supporting Information**

**S3 Table.** Effects of two week-GQ-16 treatment on body weight, food intake, cardiac mass, epididymal fat mass, inguinal fat mass and brown fat mass in male Swiss mice.

|  | Control diet | HFD + vehicle | HFD + RSG | HFD + GQ-16 |
| --- | --- | --- | --- | --- |
| Body weight (g) | 50.8±4.4 | 66.0±4.6 | 75.4±6.0^*^ | 52.0±3.8^+^ |
| Food intake (g/day) | 4.6±0.3 | 4.7±0.2 | 4.2±0.1 | 6.2±0.6^&^ |
| Cardiac mass (g) | 182.4±9.1 | 198.6±9.6 | 206.0±7.8 | 186.3±14.8 |
| Cardiac mass (mg/g BW) | 3.7±0.4 | 3.1±0.2 | 2.8±0.2 | 3.6±0.1 |
| EpiWAT mass (g) | 1.7±0.2 | 3.3±0.2^*^ | 5.0±0.6^*,#^ | 1.6±0.2^#,+^ |
| EpiWAT mass (mg/g BW) | 33.7±6.0 | 50.0±3.8^*^ | 66.8±4.8^*,#^ | 30.0±4.6^#,+^ |
| IngWAT mass (g) | 0.7±0.1 | 1.8±0.3^*^ | 2.6±0.3^*,#^ | 0.7±0.1^#,+^ |
| IngWAT mass (mg/g BW) | 14.0±1.0 | 26.6±3.1^*^ | 34.3±3.7^*^ | 14.0±2.9^#,^^+^ |
| iBAT mass (g) | 0.3±0.02 | 0.5±0.05 | 0.8±0.2^&^ | 0.1±0.02 |
| iBAT mass (mg/g BW) | 6.7±0.5 | 7.1±0.7 | 11.4±2.4 | 3.7±0.6^+^ |

Adiposity and cardiac mass were expressed as absolute values or as the ratio of tissue weight to body weight. Data are given as mean ± SEM. Statistical analysis was done using ANOVA followed by Newman-Keuls post hoc test. ^*^ *p* < 0.05 *vs* control diet that received vehicle; + *p* < 0.05 *vs* HFD that received RSG; & *p* < 0.05 *vs* all other groups, # *p* < 0.05 *vs* HFD group that received vehicle. n = 4 animals per group. epiWAT, epididymal white adipose tissue; iBAT, interscapular brown adipose tissue; ingWAT, inguinal white adipose tissue; BW, body weight; HFD, high fat diet; RSG, rosiglitazone.
